# Skimming and storage factors affect the detection of heat shock protein 70 in raw bovine milk

**DOI:** 10.3168/jdsc.2025-0759

**Published:** 2025-05-12

**Authors:** M.R.H. Rakib, V. Messina, J.I. Gargiulo, N.A. Lyons, I.N. Pathirana, P.C. Thomson, S.C. Garcia

**Affiliations:** 1 Dairy Science Group, School of Life and Environmental Sciences, Faculty of Science, The University of Sydney, Camden, NSW 2570, Australia; 2 Bangladesh Livestock Research Institute, Savar, Dhaka 1341, Bangladesh; 3 Dairy UP Program, Camden, NSW 2570, Australia; 4 NSW Department of Primary Industries and Regional Development, Menangle, NSW 2568, Australia; 5 DairyNZ, Hamilton 3240, New Zealand; 6 Department of Animal Science, Faculty of Agriculture, University of Ruhuna, Kamburupitiya 81100, Sri Lanka; 7 Sydney School of Veterinary Science, Faculty of Science, The University of Sydney, Camden, NSW 2570, Australia

## Abstract

Heat shock proteins (HSP) play a vital role in maintaining cellular function under elevated temperatures. Among these, heat shock protein 70 (HSP70) has recently been detected in bovine milk using competitive ELISA, highlighting its potential as a noninvasive marker of cellular stress and a tool for detecting heat stress (HS) in dairy cows. However, optimal management and storage conditions of milk samples for detection of HSP70 are yet to be determined. This study aimed to evaluate how different management and storage practices affect detection of HSP70 in milk samples. Samples were skimmed by centrifugation either before storage (SBS) or after storage (SAS), and then stored under different temperatures: (a) room temperature (T_1_, ∼22°C), (b) refrigerated (T_2_, 4°C), (c) frozen (T_3_, −20°C), and (d) refrigerated at 4°C with a preservative (bronopol; T_4_). Samples were analyzed every 2 d until d 15 (d 1, d 3, …, d 15) using a competitive ELISA system (sensitivity range 31.25–2,000 ng/mL) to detect HSP70 levels. Result showed that SBS samples had the smallest reduction in HSP70 levels on d 3 when stored at T_2_ or T_3_, with decreases of 4.4% and 7.9%, respectively. In contrast, SAS samples exhibited greater reductions (44.2% at T_2_ and 53.9% at T_3_). Samples stored at room temperature (T_1_) showed the greatest degradation, especially in SAS samples, whereas the use of a preservative (T_4_) did not consistently maintain HSP70 levels in either SBS or SAS samples. The HSP70 detection showed lower variability during early storage (d 1–3) across all treatments, with SBS samples demonstrating greater stability than SAS; however, variability significantly increased by d 9 in both sample types. An apparent increase in HSP70 levels was observed from d 11 to 15 in both SBS and SAS, suggesting the influence of protein stability and interactions affecting ELISA reactivity, where further research is required. Overall, samples stored as skim milk until d 3 at 4°C or −20°C preserved HSP70 levels more effectively than whole milk, with room temperature storage being the least favorable. This pioneering study explored how sample storage and management affect HSP70 detection, providing practical guidelines for farmers and processors to preserve milk sample integrity for assessment of HSP70 as indicator of cellular stress and, potentially, HS.

## Highlights

• Skimming milk before storage reduces HSP70 degradation compared with after storage.
• Skim milk stored at 4°C or -20°C preserves HSP70 levels effectively for up to 3 days.
• Storage beyond 5 days causes ~50% HSP70 degradation across all treatments.

## Graphical Abstract

**Figure:**
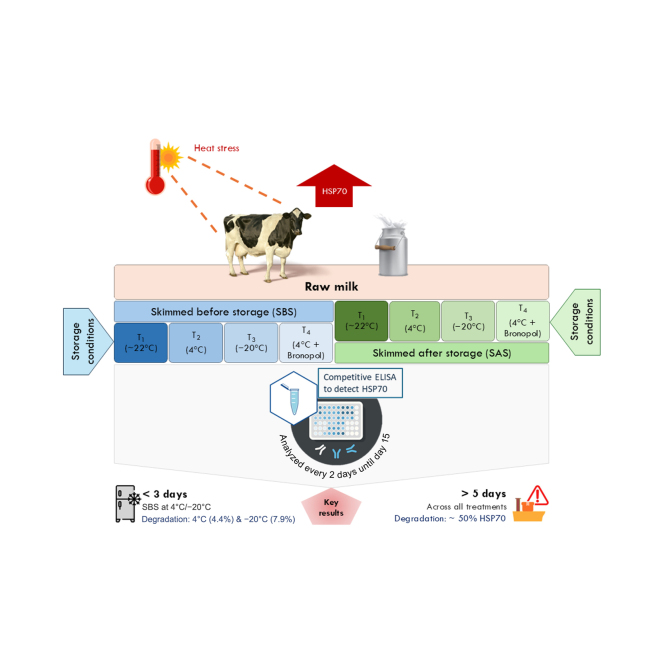
**Summary:** This study evaluated the impact of milk sample handling on the detection of heat shock protein 70 (HSP70) in bovine milk by using a competitive enzyme-linked immunosorbent assay (ELISA) system. Samples were processed as skimmed before storage (SBS) or skimmed after storage (SAS) and stored under various conditions: room temperature (~22°C; T1), refrigeration (4°C; T2), freezing (-20°C; T3), and refrigeration with preservative (4°C + bronopol; T4). Samples were analyzed every 2 days over 15 days to assess HSP70 stability. Results showed that SBS samples stored at 4°C or -20°C for up to 3 days preserved HSP70 levels with minimal degradation (4.4% and 7.9%, respectively), whereas SAS samples showed significantly higher degradation (44.2%-53.9%). Room temperature storage led to the greatest degradation, and bronopol use did not consistently maintain HSP70 levels. These findings highlight the importance of optimizing milk storage to preserve HSP70, a potential biomarker for cellular and heat stress in dairy cows.

Heat stress (**HS**) in lactating dairy cows not only reduces feed intake, milk yield, and reproductive performance, but also affects milk quality and composition, which has direct implications for dairy processing and product consistency (Becker et al., 2020; Rakib et al., 2020). Managing HS has become more complex due to the increasing number of high-producing animals, which are metabolically more active (Polsky and Von Keyserlingk, 2017), and the ongoing impact of climate change, with more frequent and severe heat events (IPCC, 2021).

Several methods for detecting HS in dairy cows have been explored, ranging from physiological and behavioral measurements to advanced applications of remote sensing and machine learning technologies (Rakib et al., 2024). Identifying practical biomarkers for HS is crucial for improving herd management and minimizing production losses. Among potential biomarkers, HSP70 is of particular interest due to its critical role in cellular stress response in dairy cattle and other livestock species, including pigs and poultry (Gaughan et al., 2013). HSP70 helps protect cells under stress by facilitating proper protein folding and preventing the aggregation of denatured proteins. By binding to misfolded proteins, it helps maintain protein homeostasis and limits the accumulation of cellular damage during HS (Mosser and Morimoto, 2004; Mayer and Bukau, 2005). Additionally, HSP70 is involved in signaling and regulatory pathways that enhance the cellular adaptive response. Its expression is upregulated through a complex network involving heat shock factors (**HSF**), element-binding proteins, and co-chaperones (Voellmy, 2004). Given its role in maintaining cellular balance and its release both within and outside cells in response to stress, HSP70 shows strong potential as an indicator of tissue-level stress (Rakib et al., 2024). However, HSP70 is not specific to HS alone; its expression can also be induced by other physiological stressors, including inflammation and disease conditions such as mastitis (Hyder et al., 2017). Understanding these influences is essential for interpreting HSP70 levels accurately and for exploring its broader diagnostic potential. Nevertheless, HSP70 potentially remains as a valuable supplementary tool for identifying heat stress or resilience in individual animals, supporting genetic selection, and informing herd management strategies (Rakib et al., 2024).

Although increases in HSP70 levels in blood and saliva have been associated with HS in cattle (Haque et al., 2012; Lamy et al., 2017), the invasiveness of sample collection can itself induce stress the animals and the process is notably labor-intensive (Lamy et al., 2017; Pathirana and Garcia, 2022), making routine application impractical for farmers. Pathirana and Garcia (2022) developed a competitive ELISA test for detecting HSP70 in milk samples, presenting promising avenues for further exploration. However, sample management factors such as storage conditions before analysis could affect the accuracy and limit the potential application of the method, yet these factors have not been quantified. For instance, if immediate analysis were required after collection, the method would be impractical on farms. Therefore, the objective of this research was to evaluate the effect of different milk management and storage practices on the detection of HSP70 in dairy cows using a competitive ELISA system.

The study was conducted at Corstorphine Dairy Farm, The University of Sydney, Camden, NSW, Australia, between August and September 2023. Milk samples were collected from 20 Holstein Friesian cows in their third lactation, with an average of 114 DIM. All cows grazed on annual ryegrass-based pasture and showed no signs of health issues such as mastitis or lameness, minimizing potential confounding effects on HSP70 levels. Milk sampling was performed on the first day of the experiment during a single, scheduled afternoon milking session between 1300 and 1400 h, with samples collected from all 4 quarters of each cow. According to per-minute environmental data from the Australian Bureau of Meteorology, ambient temperature during this sampling period ranged from 20.4°C to 22.6°C, and relative humidity ranged from 34% to 39%. These consistent environmental conditions minimized the potential influence of acute thermal stress on HSP70 levels across animals on the sampling day. Moreover, the ∼22°C ambient temperature also aligned with the designated room temperature condition used in this experiment, which was selected to reflect typical sample handling environments encountered in laboratories or farm-side settings, particularly in moderate climates.

Approximately 250 mL of milk per cow was collected into rubber snap-seal sample bottles. Milk yield (L/d) was recorded using WB Ezi-Test milk meters (Tru-Test, New Zealand). Milk composition, including fat, SNF, protein, and lactose, was analyzed using a MASTER ECO ultrasonic milk analyzer (Milkotester Ltd., Bulgaria) following the manufacturer's instructions. Each milk sample was then divided into eight 15-mL subsamples (Figure 1). Subsamples were handled as follows: skimmed before storage (**SBS**): fat was removed via centrifugation (1,000 × *g* for 10 min at 4°C) immediately after collection; and skimmed after storage (**SAS**): fat was removed via centrifugation (1,000 × *g* for 10 min at 4°C) after the samples had been stored under specified conditions.

**Figure 1:**
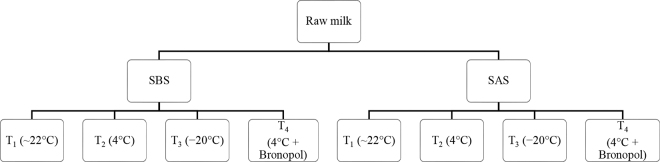
Experimental design of storage conditions to detect HSP70 in raw bovine milk. SBS = skimmed before storage; SAS = skimmed after storage; T_1_ = room temperature (∼22°C); T_2_ = refrigerated (4°C); T_3_ = frozen (−20°C); T_4_ = adding bronopol preservative and refrigerated (4°C).

Subsequently, samples were subjected to different storage conditions: (a) room temperature (**T_1_**, ∼22°C), (b) refrigerated (**T_2_**, 4°C), (c) frozen (**T_3_**, −20°C), and (d) refrigerated at 4°C (**T_4_**) with added preservative (bronopol). Each subsample (15 mL) was stored in a Falcon tube placed inside a labeled plastic bag. Samples were analyzed every 2 d until d 15 (d 1, d 3, …, d 15). Before ELISA analysis, frozen samples were thawed completely at room temperature, ensuring consistency in the measurement process.

The concentration of HSP70 in milk samples, analyzed in duplicate, was measured using a competitive ELISA system, exactly as previously described by Pathirana and Garcia (2022). In brief, 96-well polystyrene microtiter plates (Nunc Immuno Polysorp, Rochester, NY) were coated with recombinant human HSP70 (ADI-NSP-555, Enzo Life Sciences, Farmingdale, NY) and incubated overnight at 4°C. Samples and standards were pre-incubated with a monoclonal anti-human HSP70 antibody (ADI-SPA-810, Enzo Life Sciences) overnight at 4°C and then added to the blocked wells. After incubation and washing, a goat anti-mouse IgG antibody conjugated with horseradish peroxidase (HRP, BML-SA204, Enzo Life Sciences) was applied. The color reaction was obtained using 3,3′,5,5′-tetramethylbenzidine (TMB, Sigma, St. Louis, MO) substrate, and the reaction was stopped with 1 *N* hydrochloric acid. Absorbance was measured at 450 nm using an ELISA plate reader (MR9600, Accuris, NJ). The intra- and inter-assay coefficients of variation were determined to be 8.1% (n = 3) and 16.9% (n = 3), respectively. The minimum detection limit of the assay was 31.25 ng/mL, and the detection was reliable in the range from 31.25 to 2,000 ng/mL.

Data processing, statistical analysis, and visualization were performed using R (version 4.3.2; https://www.r-project.org/) statistical software. Descriptive statistics, including the mean, SD, coefficient of variation %, and the range (minimum and maximum values), were calculated for HSP70 and key variables such as milk yield, fat, SNF, protein, and lactose (Table 1). Pearson correlation coefficients and corresponding *P*-values were calculated to investigate the relationships between HSP70 concentration (ng/mL), milk yield (L/d), and milk composition (fat, SNF, protein, and lactose). A linear mixed model (*lme4* package) was used to compare the effects of sample handling methods (SBS and SAS), storage conditions, storage time, and their interactions on HSP70 levels. Animal ID was included as a random effect to account for multiple observations per sample. Pairwise comparisons of estimated marginal means were conducted within each combination of sample handling method, storage condition, and storage time using the *emmeans* and *multcomp* packages, with Sidak adjustment applied for multiple testing. Model residuals were assessed for normality and heteroscedasticity, and extreme outliers were removed before final analysis. Moreover, Levene's test was performed to evaluate homogeneity of variances across sample handling methods × storage condition × storage time combinations. In addition, generalized least squares (**GLS**) modeling was applied to compare models assuming constant residual variance with those allowing group-specific variances using the *varIdent* function from the *nlme* package. Model comparison was conducted using likelihood ratio tests. All tests were performed at a significance level of *P* < 0.05 with 95% CI reported where applicable.

**Table 1:** Descriptive statistics of the variables from the dataset at d 1

Variable	Mean	SD	CV (%)	Minimum	Maximum
HSP70 concentration (ng/mL)	210.08	17.37	8.27	177.08	248.44
Milk yield (L/d)	32.75	10.63	32.47	12.50	51.25
Milk composition (%)					
Fat	3.99	1.51	37.94	1.30	6.00
SNF	8.87	0.48	5.37	7.90	9.90
Protein	3.22	0.17	5.17	2.90	3.60
Lactose	4.84	0.26	5.35	4.30	5.40

As shown in Table 1, HSP70 levels averaged 210.08 ng/mL, with a moderate variability in d 1 (CV = 8.27%). Milk yield had a high variability (CV = 32.47%), whereas milk composition variables showed a range of variation, with fat percentage having the highest CV (37.94%), whereas SNF, protein, and lactose percentages exhibited more consistency, with CV around 5%.

Additionally, the Pearson correlation coefficients between HSP70 concentration (ng/mL), milk yield (L/d), and different milk composition parameters (fat, SNF, protein, and lactose) were calculated in d 1. HSP70 exhibited a negative correlation with milk yield (−0.41), suggesting that higher HSP70 levels are associated with reduced milk production. However, no significant correlations were observed between HSP70 concentration and milk composition (fat, SNF, protein, lactose). These results indicate that although HSP70 concentration is linked to milk yield, it is not directly correlated with milk composition.

The main effects of sample handling methods, storage conditions, and storage time, together with their 2-way and 3-way interactions, were all statistically significant (*P* < 0.0001). Given the significant interactions observed, pairwise comparisons were interpreted within each day × handling method × storage condition combination. On d 3, SBS samples showed the least reduction in HSP70 levels, with decreases of 4.4% for T_2_ (4°C), 7.9% for T_3_ (−20°C), 21.3% for T_1_ (room temperature, ∼22°C), and 57.9% for T_4_ (4°C with preservative), compared with d 1 (210.08 ng/mL; Figure 2). In contrast, SAS samples on d 3 exhibited larger reductions, with 44.2% for T_2_, 49.7% for T_4_, 53.9% for T_3_, and 56.6% for T_1_, indicating that skim milk stored at T_2_ (4°C) was most effective in preserving HSP70 levels until d 3, followed by frozen storage at T_3_ (−20°C). Samples stored at room temperature (T_1_) showed a higher degradation, particularly for SAS, whereas the use of a preservative (T_4_) did not consistently maintain HSP70 levels in either SBS or SAS samples.

**Figure 2:**
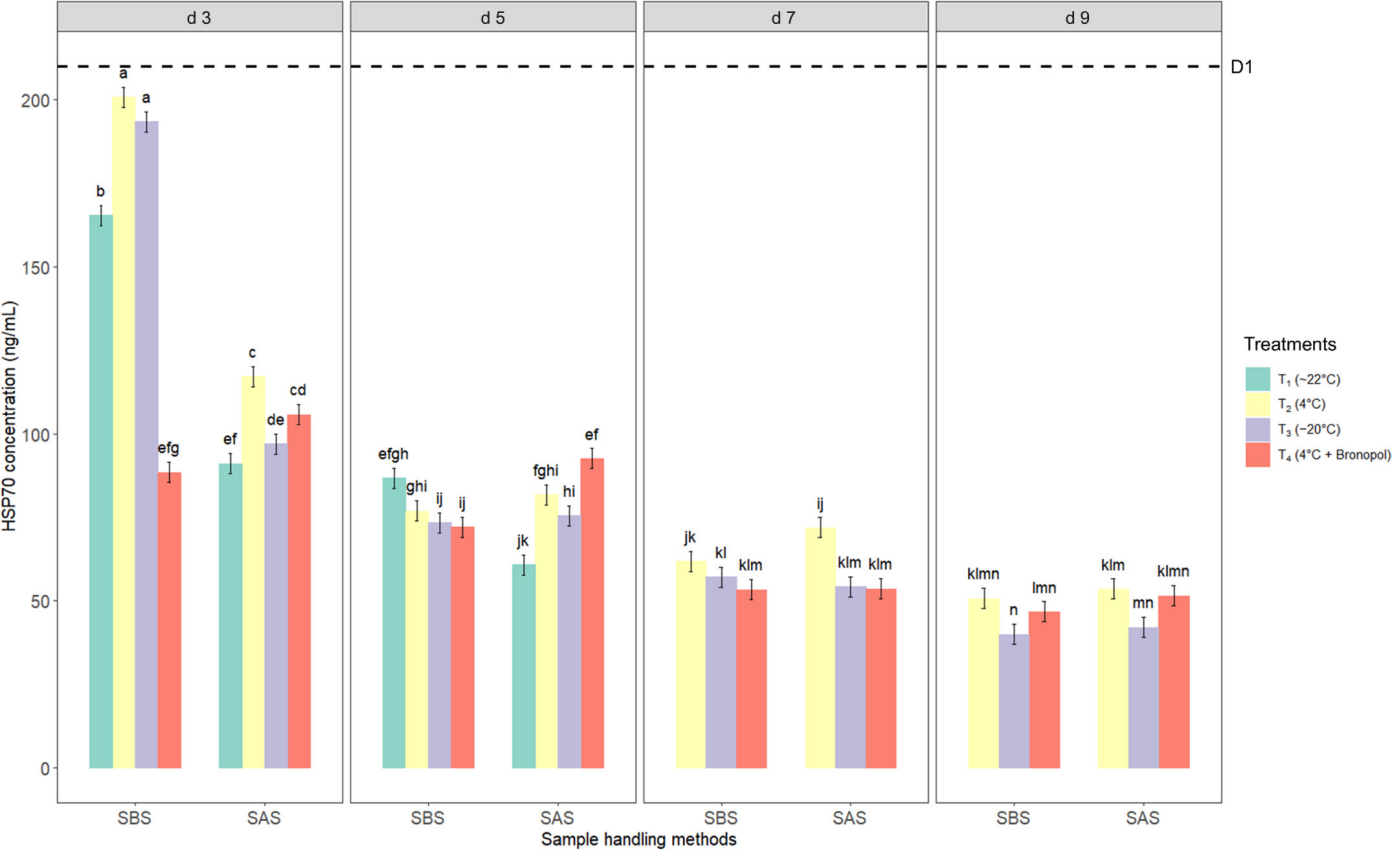
Effect of sample handling methods, storage conditions, and storage time on HSP70 concentration (ng/mL) in skim milk before and after storage. Vertical lines represent the SEM. Bars with different letters indicate significant differences (*P* < 0.05) based on Sidak-adjusted pairwise comparisons within each combination of sample handling method, storage condition, and storage time. The dashed line represents expression of HSP70 at d 1 without storage effect.

After d 3, a decrease in HSP70 levels was observed in both SBS and SAS samples, with further reductions from d 3 to 9 across all storage conditions. However, after d 5, HSP70 was no longer detected in T_1_ samples (room temperature, ∼22°C) due to sample spoilage. The HSP70 levels in SBS samples remained relatively higher than in SAS samples until d 3, after which both handling methods showed a similar trend, suggesting that centrifugation may help to preserve protein stability in the early stages of storage. From d 11 to 15, an increase in measured HSP70 levels was observed in both handling methods (data not shown), indicating the influence of protein stability and interactions affecting ELISA reactivity, warranting further research.

The CV of HSP70 ranged from 8.27% to 40.95% across samples, with lower variability observed in the earlier days (d 1–3) for most treatments, indicating more consistent HSP70 detection during this period. In SAS samples, variability ranged from 10.3% to 12.7% on d 3 across different treatments but increased significantly to 36.43% by d 9. The SBS samples were generally more stable in shorter treatments, with the lowest CV of 8.26% observed on d 1; however, variability increased to a range of 30.55% to 32.52% by d 9 for different treatments.

Similarly, Levene's test revealed significant heterogeneity in HSP70 variability across all combinations of sample handling method, storage condition, and storage time (*F* = 7.99, *P* < 0.001), as well as for each factor individually (all *P* < 0.001). The GLS modeling further confirmed that residual variance differed significantly across these groups: the model allowing group-specific variances to fit substantially better than the constant-variance model (likelihood ratio = 806.1, *P* < 0.001), which suggest that both sample handling methods and treatment duration significantly influence the stability and detectability of HSP70.

Previous research on the effects of sample handling, storage conditions, and time on ELISA results is limited but provides valuable insights. Charlier et al. (2005) demonstrated that storing whole milk samples for up to 4 d at 4°C did not significantly affect the detection of gastrointestinal nematode (*Ostertagia ostertagi*) infections in adult cows using an indirect ELISA, compared with skim milk samples. Additionally, bronopol, a common preservative used in milk sample collection, has generally been reported to have no significant impact on ELISA performance in certain assays (Sweeney et al., 1994; Sanchez et al., 2002). For example, Sanchez et al. (2002) found high repeatability between preserved and nonpreserved samples stored for up to 42 d at 4°C using an *O. ostertagi* ELISA, and da Silva et al. (2017) similarly reported stable results in bronopol-treated samples analyzed for pregnancy-associated glycoproteins. However, in this study, bronopol-preserved samples consistently showed lower HSP70 concentrations compared with samples stored at 4°C without preservative. A potential explanation for this is that bronopol generates reactive oxygen species and oxidizes thiol groups to disulfides. As HSP70 contains cysteine residues with reactive thiol groups, bronopol may alter its structure, potentially affecting antibody recognition and detection in ELISA (Shepherd et al., 1988; Zhang et al., 2022). This aligns with findings from other ELISA-based studies suggesting that bronopol can interfere with specific assay components. Thomas et al. (2016) observed altered ELISA readings for C-reactive protein in bronopol-treated milk, whereas Wilson et al. (2012) reported reduced detection of anti-bovine viral diarrhea antibodies in bronopol-preserved milk stored at room temperature. Klintevall et al. (1991) further demonstrated that excessively high concentrations of bronopol led to reduced ELISA absorbance values, likely due to interference with colorimetric detection rather than analyte degradation. These studies support the interpretation that bronopol may interact with the ELISA system possibly affecting antibody binding or enzymatic activity resulting in lower apparent concentrations. This highlights the need for further validation of preservative compatibility in analyte-specific ELISA and cautions against assuming uniform preservative behavior across assay types.

From d 11 to 15, both SBS and SAS samples exhibited an unexpected rise in HSP70 levels, suggesting that prolonged storage might influence protein stability and interactions. One possible explanation is the degradation of proteins, which could release HSP70 previously bound to other molecules, thereby increasing its detectable levels. Another possibility is the denaturation of proteins, which could lead to cross-reactivity with the test assay, as hypothesized by Wynands et al. (2017). These authors evaluated the effect of storage temperature and the time from milk sample collection to laboratory analysis on pregnancy-associated glycoproteins levels using ELISA for pregnancy diagnosis in dairy cows. While such increases may not significantly affect routine diagnostic tests for producers, they could have implications for research-focused sample storage and the interpretation of results.

To the best of our knowledge, this is the first study to investigate the effects of sample storage and skimming on HSP70 detection in bovine milk, providing important insights into ways for minimizing its degradation. Our findings reveal that storing skim milk refrigerated at 4°C or frozen at −20°C until d 3 maximizes protein stability and minimizes protein degradation allowing detection of HSP70 with a competitive ELISA system. In contrast, room temperature storage decreased stability of HSP70 leading to protein degradation process, especially in SAS samples. The centrifugation process in SBS samples appeared to enhance protein stability, minimizing HSP70 degradation process. In contrast, bronopol-preserved samples showed lower HSP70 levels compared with those stored at 4°C without preservative, suggesting a potential interaction between the preservative and HSP70, though further research is needed to confirm this. These results provide practical guidelines for farmers and processors to preserve milk sample integrity for assessment of HSP70 as indicator of cellular and heat stress.
